# Defining the Role of the Pharmacy Technician and Identifying Their Future Role in Medicines Optimisation

**DOI:** 10.3390/pharmacy5030040

**Published:** 2017-07-15

**Authors:** Melanie Boughen, Jane Sutton, Tess Fenn, David Wright

**Affiliations:** 1 School of Pharmacy, University of East Anglia, Norwich NR4 7TJ, UK; d.j.wright@uea.ac.uk; 2 Department of Pharmacy and Pharmacology, University of Bath, Bath BA2 7AY, UK; j.sutton@bath.ac.uk; 3 Association of Pharmacy Technicians, Birmingham B1 1BD, UK; president@aptuk.org

**Keywords:** pharmacy technician, medicines optimisation, pharmacist, hospital pharmacy, community pharmacy, patient-centred

## Abstract

Background: Traditionally, pharmacy technicians have worked alongside pharmacists in community and hospital pharmacy. Changes within pharmacy provide opportunity for role expansion and with no apparent career pathway, there is a need to define the current pharmacy technician role and role in medicines optimisation. Aim: To capture the current roles of pharmacy technicians and identify how their future role will contribute to medicines optimisation. Methods: Following ethical approval and piloting, an online survey to ascertain pharmacy technicians’ views about their roles was undertaken. Recruitment took place in collaboration with the Association of Pharmacy Technicians UK. Data were exported to SPSS, data screened and descriptive statistics produced. Free text responses were analysed and tasks collated into categories reflecting the type of work involved in each task. Results: Responses received were 393 (28%, *n* = 1380). Results were organised into five groups: i.e., hospital, community, primary care, General Practitioner (GP) practice and other (which included HM Prison Service). Thirty tasks were reported as commonly undertaken in three or more settings and 206 (84.7%, *n* = 243) pharmacy technicians reported they would like to expand their role. Conclusions: Tasks core to hospital and community pharmacy should be considered for inclusion to initial education standards to reflect current practice. Post qualification, pharmacy technicians indicate a significant desire to expand clinically and managerially allowing pharmacists more time in patient-facing/clinical roles.

## 1. Introduction

Pharmacy technicians in the UK (except Northern Ireland) have been registered with the General Pharmaceutical Council (GPhC) since July 2011. National registration for pharmacy technicians in other countries is not currently a requirement.

Pharmacy technicians, also known as dispensary technicians in Australia, have traditionally worked alongside pharmacists in community and hospital pharmacy settings and have been based primarily in the pharmacy dispensary. Described as ‘a vital part of the pharmacy team’, their primary role has been the preparation and supply of medicines and healthcare products, often with additional advice and guidance [1] (p. 10). In addition to the supply of medicines via prescription, the pharmacy technician role has included production and provision of aseptically prepared medicines, extemporaneous medicines preparation and supply of medicines for clinical trials.

Increasingly, pharmacy technicians are undertaking more generic medicines management based roles and as a result of Audit Commission’s publication ‘A spoonful of sugar, Medicines Management in NHS Hospitals’ the last 15 years has seen significant change in the role of the pharmacy technician [2]. The main recommendations for pharmacists to work more closely with patients and provide clinical services resulted in a significant transfer of responsibility to pharmacy technicians. Within the UK hospital pharmacy environment, it is now not unusual for pharmacy technicians to manage the pharmacy dispensary and for them to work alongside pharmacists on the ward to ensure that tasks are undertaken using the most appropriate skills mix. New key roles for hospital pharmacy technicians include the assessment of patients’ own drugs for use during a hospital stay, undertaking medicines reconciliation for patients on admission, supplying medicines to cover the duration of a patient stay and for the period immediately after their discharge.

The introduction of pharmacists into primary care led to working with general practitioners, advising on the evidence-based use of medicines, medication safety and managing prescribing budgets. This work led to the creation of the primary care pharmacy technician who was again required to support the pharmacist in this role, and again this ensured that the person with the most appropriate skills is used for each task. Supported by a significant contract change in 2005, the government’s vision for the role of the community pharmacist has equally changed from the supply of medicines to the provision of patient care [3,4]. United Kingdom law currently prevents pharmacists from leaving the pharmacy for significant periods of time and therefore pharmacy technicians have not been able to assume responsibility for all activity within the dispensary in the same manner as that seen in the hospital setting. Community based pharmacy technicians are frequently used to provide elements of clinical and public health based services in collaboration with the community pharmacist. The advent of Healthy Living pharmacies where the accessibility of the community pharmacy was seen as providing an excellent opportunity to promote health, was perhaps the first time that pharmacy staff were recognised for their contribution to patient care as their ability to engage with the general public was seen as pivotal to the success to the service [5]. 

With these expanding and new patient-facing roles across all sectors, greater autonomy was essential to allow pharmacy technicians to make professional decisions. Represented professionally by the Association of Pharmacy Technicians (UK) (APTUK) since 1952 [6], pharmacy technicians were not regulated. In 2001, the Audit Commission recommended to the then pharmacy regulator, the Royal Pharmaceutical Society of Great Britain (RPSGB), that they should consider the formal registration of pharmacy technicians. Voluntary registration was introduced in 2005 by the RPSGB and mandatory national registration introduced in 2011 (Pharmacy Order 2010) by the newly formed regulator General Pharmaceutical Council (GPhC). 

However, there is a paucity of research into the current roles and responsibilities of pharmacy technicians and little is known about the tasks they actually perform as part of their day-to-day work. To date, research on the roles of Pharmacy technicians in the UK relates to commissioned research for national enquiry with Schafheutle et al. [7], exploring the quality of the initial training and education for pharmacy technicians delivered by providers and Rosado et al. [1], presenting an analysis of Initial education and training standards for pharmacy technicians and their fitness for purpose. Post qualification education was not within the scope of either project.

As a rapidly developing profession, the aim of this study was to capture the current roles of pharmacy technicians, and identify how their future role will contribute to medicines optimisation.

## 2. Materials and Methods 

Approval for the study was received from the University of East Anglia Faculty of Medicine and Health Sciences Research Ethics Committee on 15th April 2016 reference number: 20152016-75.

### 2.1. Content

An online survey of pharmacy technician’s views about their roles and training was administered through the online survey software SurveyMonkey™. The survey consisted of thirty questions, including demographic details, including age, gender, length of time working in pharmacy and work setting etc. The survey also included free text questions to capture pharmacy technician opinions on their current roles, future roles and training.

### 2.2. Design

We aimed to recruit up to 500 Pharmacy technicians to complete the survey from a variety of pharmacy roles, e.g., accredited checking, medicines management; from different pharmacy settings including community pharmacy, secondary care, pharmaceutical industry; and backgrounds, e.g., place of training, age and gender, as a sample size of 500 provides 95% confidence intervals of ±4%, around a 30% and 70% response to a question. An online survey was administered using a questionnaire especially designed for the purpose with questions devised in consultation with board members of the APTUK. The questionnaire used for the survey aimed to explore the perceptions of Pharmacy technicians of:

- Current role of pharmacy technicians
- Potential role of pharmacy technicians
- Current training of pharmacy technicians
- Training needs of pharmacy technicians to fulfil an extended role
- Barriers and facilitators to extending the role of pharmacy technicians
- Working as part of a multidisciplinary team and perceived team members

The questionnaire also gathered information about:

- Demographics, e.g., age, gender, length of time working/qualified etc.
- Geographical differences in the work locations of technicians, e.g., urban vs rural
- Organisational differences in the work settings, e.g., size, type

### 2.3. Pilot

To ensure face validity and rigour to the study, an initial pilot study of 6 pharmacy technicians resulted in some changes being made to the wording of some questions to make their meaning clearer. The pilot work also identified how long the questionnaire would take to complete so that this could be indicated in the participant information sheet. The responses to the questionnaires for the pilot study were not used in the main study as some changes were made to the questionnaire as a result.

### 2.4. Dissemination

Recruitment took place between 17 April 2016 and 12 June 2016 in collaboration with APTUK, with the president of the APTUK acting as gatekeeper to potential participants. The researchers did not have access to the names and addresses or any other personal information of the APTUK members who were invited to take part in the research, nor did the APTUK divulge any personal information about their members. An email was sent by APTUK to all of their members (approximately 6% of the total number of Pharmacy technicians in the UK (23,150)). This provided a pool of potential participants of 1380 and it was anticipated that we would achieve a 30–40% response rate (400–600 responses). As this was the first survey of its kind of UK pharmacy technicians, we took a conservative estimate as we were unsure of the level of engagement of the profession, therefore, our anticipated response rate could only be based on the advised response rate of previous surveys conducted by APTUK. We sought to recruit participants from different pharmacy settings across the UK. The email sent to APTUK members invited them to take part in the study. Information and a copy of the email were also placed on the APTUK website as open access and using social media via Twitter™ and Facebook™ and community pharmacy networks to capture pharmacy technicians who were non-APTUK members. In addition to our dissemination strategy, to maximize the response rate, the email was also sent to senior managers of community pharmacy organisations including Pharmacy Voice and the National Pharmacy Association and asked to disseminate to community pharmacies. Reminder emails were sent out twice and a verbal reminder was given during the President’s address at the annual APTUK conference. Tweeting and retweeting occurred on a regular basis.

At the end of the email, an information sheet informed potential participants how their information would be used and that participation was voluntary. It also reassured them of confidentiality and that if they decided to take part, they were free to change their mind later without reason, provided this was before they submitted their response as the questionnaire was anonymised from the start and would therefore be unidentifiable. Prospective participants were also advised of the estimated time it would take to complete the questionnaire and that their decision to participate or not would not affect their employment in any way.

### 2.5. Data Anaylsis

The analytic process was both descriptive and explanatory. Data were exported from SurveyMonkey™ and entered into SPSS statistical software for Windows version 20 and following data screening, descriptive statistics were produced for participants’ characteristics and their descriptions of their roles, career development, multi-disciplinary team work and training. Analyses were carried out by JS with interpretation of the findings being the responsibility of all the members of the project team.

The responses to free text questions were analysed using content analysis to provide a complete picture of the participants’ perceptions using their own words and comparing them across settings and organisations [8]. Two of the researchers agreed on the allocation and categorisation of the classification of the tasks and the tasks respondents reported carrying out were listed and then collated into categories that reflected the type of work involved in each task. Expert Pharmacy technicians on the research team carried out this section of the analysis.

## 3. Results

Four hundred and seventy-two pharmacy technicians responded to the questionnaire (34%). The data were exported from SurveyMonkey™ and converted to SPSS for Windows version 20. The data were screened and it was found that 79 respondents did not add responses to any of the questions. These were removed from the database resulting in data derived from 393 (28%) pharmacy technicians.

The results were organised according to pharmacy setting of hospital, community (retail pharmacy), primary care (working in medicines management roles in Clinical Commissioning Groups in England, Health Boards in Wales and Scotland providing medicines management support to prescribers, practice staff and patients in a primary care setting) and GP Practice (at the time of this study this related to dispensary based pharmacy technicians rather than practice based). Smaller, or more specialised settings, e.g., Education, HM Prison Service, Industry, are group together as ‘other settings’. Not all respondents were required to answer all questions and some questions asked respondents to indicate more than one answer. For this reason, the number of responses to each question may not add up to the total number of respondents from each pharmacy setting.

### 3.1. Demographics

Table 1 summarises the demographic data provided by all respondents. Data showed that 254 (64%) of respondents work in hospital pharmacy, 71 (18%) in community pharmacy, 41 (10%) in primary care, 28 (7%) in General practice and 50 (13%) work in ‘other’ settings. The majority of respondents (85%) were female. The area of work with the highest number of respondents was the ‘town’ setting (41%) and the least was the ‘rural’ setting (4%). The majority of respondents (88%) work between 31–40 h per week, and 73 (19%) pharmacy technicians work over 40 h per week. Some pharmacy technicians’ work in more than one setting, therefore, demographic data on work setting exceeds the number of actual forms returned.

### 3.2. Tasks Undertaken by Pharmacy Technicians

A total of 30 tasks were reported by respondents as commonly undertaken in three or more settings. Community and hospital pharmacy technicians responded as undertaking all 30 (100%) tasks listed, pharmacy technicians working in primary care commonly undertake 19 (63%) of all tasks, GP practices, 23 (74%) of tasks and ‘other’ settings 14 (46%) of tasks. Within ‘other’, some pharmacy technicians may undertake more tasks than reported if their setting allows, e.g., HM Prison. Table 2 provides a list of the tasks that the respondents stated that they undertake following qualification, and which sector commonly undertakes which tasks. Technical tasks refers to tasks that are process driven and do not directly involve the patient, clinical tasks refers to tasks which relate to specific treatments or the healthcare of a patient, and management and training relates to tasks that involve the supervision, management and training of others within the organisation.

The tasks reportedly carried out by community pharmacy technicians are less extensive than those of hospital pharmacy technicians and vary in their focus. The clinical tasks are fewer than those carried out by hospital pharmacy technicians but include involvement in essential, advanced and local services, such as smoking cessation, assisting with Medicines Use Reviews (MURs) and supervised methadone consumption.

Tasks undertaken in the primary care sector are more diverse. Technical tasks include data analysis and writing reports on prescribing/incidents/usage and wastage as well as data analysis for medicines management incentive schemes, medicines switches and investigating and reviewing incident reporting. Clinical tasks include reviewing patients’ medicines in their own homes, and medicines management in nursing homes. Management tasks including staff management and dealing with complaints as well as training and development responsibilities was seen in all settings.

Pharmacy technicians in GP practices reported undertaking tasks that were similar to those undertaken in community pharmacy except for the commissioned services.

Pharmacy technicians working in ‘other’ settings reported undertaking tasks from all four main categories, covering many of the tasks described previously in hospital, community and primary care.

Table S1 provides a complete list of all tasks identified and in which sector they are undertaken.

#### 3.2.1. The Future Role of Pharmacy Technicians

**Figure 1:**
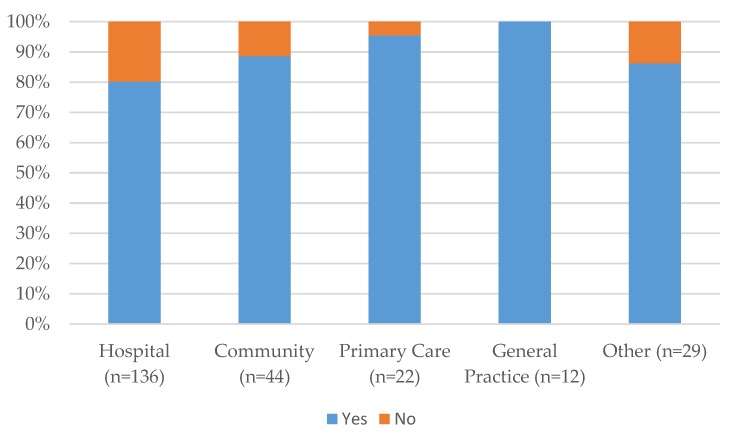
Results by pharmacy sector of responses to expanding the role of the pharmacy technician.

A total of 243 (61.8%, *n* = 393) pharmacy technicians responded when asked if they would like to expand their role. The results across all sectors imply that the majority of pharmacy technicians want to expand their role 206 (84.7%, *n* = 243) with the highest percentages being in primary care, 21 (95.5%, *n* = 22) and community pharmacy 39 (88.6%, *n* = 44). As opposed to 37 (15.2%, *n* = 243) across all sectors who said they would not want to expand their role. The results are presented sector by sector in Figure 1. 

#### 3.2.2. How Could the Pharmacy Technician Role Expand?

Respondents were asked to comment on how they thought the role of the pharmacy technician could be expanded.

Most hospital respondents saw the future role as one with a greater emphasis on clinical care:

“I think that in the future, clinically based pharmacy technicians will continue to take on more and more clinical work, taking part in running clinics and ever more detailed medication reviews…for highly skilled and specialised technicians to be involved in prescribing.”

In community pharmacy, a desire to become involved in more patient facing roles was expressed:

“I don't feel my skills, training and experience are utilised at all within my current role. I could be more involved in targeting potential MUR patients and NMS [New Medicines Service] patients. If I was given the opportunity to continue studying, then I would be of far more use to the company.”

“I would like to be given more responsibility, and put in to practice what I have learnt.”

Intermediate care and working across boundaries was seen as somewhere that pharmacy technicians in primary care may play an increasing role:

“With an emphasis on moving patient care away from hospitals, the intermediate care setting is a key area for technicians to excel”.

“I feel that my role could be expanded by getting much more involved with community pharmacies and hospital pharmacies to ensure seamless care on discharge.”

In the GP practice setting, pharmacy technicians expressed the benefits through extending integration with the practice

“Could be further integrated into GP practice to include a seamless service with medicines from home to hospital.”

Pharmacy technicians want to take on more specialist, leadership and management roles previous done by pharmacists.

“... . Technicians should also take over operational higher level roles within pharmacy dept. These do not need to be done by pharmacists anymore.”

## 4. Strengths and Limitations

Obtaining data from 393 pharmacy technicians considering the length of the survey is good given the target of 500, but a response rate of 34% (reduced down to 28%) could still be perceived by some as low when looked at in isolation and when broken down into separate sectors, are left with much smaller numbers. Interestingly, there was a considerably smaller number of community pharmacy responses compared to hospital pharmacy responses, when community pharmacy has a significantly larger number of pharmacy technicians. Whilst the survey was sent to senior managers of community organisations, we do not know whether it was disseminated to the pharmacy technicians. What we do know is that the APTUK membership is made up of a significantly larger proportion of hospital based pharmacy technicians, and this is reflected in the results. The larger proportion of pharmacy technicians working in rural settings implies that these are pharmacy technicians working within dispensing GP practices.

The majority of hospital, community and primary care respondents were female, and under the age of 40 reflecting the willingness of this group to engage with professional activities.

## 5. Discussion

This is the first nationwide survey of pharmacy technicians in the UK to describe the range of activities undertaken and their beliefs about their future roles.

These results suggest that the significant variety and number of tasks undertaken by pharmacy technicians and the sectors in which they are undertaken makes defining the role of the pharmacy technician difficult as sectors and settings are diverse. However, with the initial education and training standards for pharmacy technicians in the UK currently under review by the GPhC, the results show the commonality seen between roles within the different settings and that there is a core set of skills that should be included as part of initial education and training, some which are existing and some which are new. These would lay the foundation of knowledge and skills to 21st century pharmacy practice, and should therefore be considered for inclusion in the new standards [9].

The three sectors that have the dispensary as a base: community, hospital and GP practice, all indicate that they undertake final accuracy checking of prescribed medicines, and this activity should therefore be considered for inclusion in the initial education and training standards review. It is also clear that pharmacy technicians are no longer confined to the dispensary with pharmacy technicians regularly carrying out activities all of which support the medicines optimisation agenda. Across all sectors, they are involved directly in patient counselling, involvement in undertaking medicines use reviews in community pharmacy, or drug history taking and medicines reconciliation in hospital and primary care pharmacy.

The people with whom pharmacy technicians now communicate has changed, and involves those both internal and external to organisations, for example, patients, colleagues and multi-disciplinary teams, and other service users such as care or nursing homes. All of these examples contribute to the patient’s experience and patient-centred care, whether directly through patient counselling on their medicines and/or lifestyle advice, or indirectly, ensuring that the correct medicines are available for the patient irrespective of the patient’s setting. The level of communication skills required needs to be learned from the early stages of pharmacy technician training to enable the trainee to acquire the confidence and ability as they reach entry level from which they can then develop further as they gain experience post registration.

With many pharmacy technicians suggesting that they feel underused, there is an overwhelming desire to expand existing roles. This is more noticeable in primary care, community pharmacy and GP practice where there is less evidence of education and development infrastructure compared to the hospital environment.

Although many view role extension as developing the clinical role, others see role extension as an increase in managerial responsibilities. Irrespective of whether clinical or managerial, assuming these activities previously undertaken by pharmacists while remaining within the boundaries of the pharmacy technician profession, allows pharmacists to spend more time in patient-facing or clinical roles and extending patient services.

Much of what has been discussed on the changes in pharmacy technician practice in hospital and community pharmacy is reinforced by two studies published in 2016. Specifically in relation to hospital pharmacy, the independent report for the Department of Health, Lord Carter of the Coles (2016) suggests ways of improving efficiency in the NHS; how close working with patients and the multidisciplinary team is needed to deliver medicines optimisation, and the need for clinical pharmacy technicians to spend more time on clinical pharmacy services than on other activities [10].

In community pharmacy, discussions on supervision are ongoing and a more recent report published since this research was undertaken, the Community Pharmacy Clinical Services Review (2016) commissioned by the Chief Pharmaceutical Office for NHS England identifies that pharmacy technicians are a regulated professional staff group who are an essential part of the pharmacy team and have the scope to take on new roles; further suggesting that a shift in the balance of work which allows pharmacy technicians to take over the day to day management of the dispensary [11,12]. Primary care is not to be excluded from extended roles as with developments seen within the whole pharmacy profession, primary care pharmacy technicians can be found working at the interface, providing integrated services during domiciliary visits, working with nursing and care homes, as well as being located within GP practice settings.

A further pathway that crosses all sectors is the support that pharmacy technicians are providing to the education, training and development of others. With an emphasis on work-based training, pharmacy technicians have been able to expand their role in another direction. Pharmacy technicians from all sectors appear to be involved in training, although this can vary from training pharmacy/dispensary assistants, or someone new to the workplace, while others provide more formal practice-based training and teaching to pre-and post-registration pharmacists and pharmacy technicians, and to those extending their roles into medicines management. Delivering high standard and consistent training requires a specific set of skills, and training for trainers should be available for anyone that works in a training capacity.

There are however some barriers when considering the future roles of pharmacy technicians in the UK. As well as funding to support training, a significant barrier lies with management, where respondents suggested that there is a lack of awareness of the capabilities of pharmacy technicians and advancement is stifled; on the other hand, it is clear that where the roles and capabilities of pharmacy technicians are understood by management and organisations the benefits to patient services are recognised, and role expansion and development facilitated.

## 6. Future Research

Our research has shown that there is the potential and the will on the part of pharmacy technicians in the UK to expand their role. Future research could include:

- Full analysis of the tasks, skills and training required by pharmacy technicians
- Identification of the professional attributes required by pharmacy technicians
- Examination of the nature and quality of mentoring of pharmacy technicians in training
- The relationship between pharmacists and pharmacy technicians and how this might be improved through greater understanding of each other’s roles
- The management culture in pharmacies and how this can be enhanced to better utilise pharmacy technicians

## 7. Recommendations

The recommendations below have been drawn from the results of this research and will require significant collaboration between pharmacy staff and other members of the MDT. They also require the full support of the regulators and the pharmacy professional leadership bodies to work together to promote high levels of professionalism, education and career development of the pharmacy team:

- Review the education and training needs of pharmacy technicians in light of the roles and activities now commonly undertaken and the identified new knowledge and skills which need to be incorporated into pre-registration training
- Review post-registration education and training to ensure that opportunities exist which enable the preparation of pharmacy technicians for the wide variety of roles
- Develop a post-registration career framework to provide a career structure for registered Pharmacy technicians

## Figures and Tables

**Table 1:** Summary of demographic data.

Responses per Pharmacy Sector					
	**Hospital**	**Community**	**Primary Care**	**GP Practice**	**Other**
**Gender:**	*n =* 254	*n =* 71	*n =* 41	*n =* 28	*n =* 50
Female	210 (82.7%)	61(86%)	37 (90.2%)	26 (92.9%)	44 (88%)
Male	44 (17.3%)	10 (14%)	4 (9.8%)	2 (7.1%)	6 (12%)
**Age:**	*n =* 252	*n =* 70	*n =* 40	*n =* 27	*n =* 50
Under 20	0	1(1.4%)	0	0	0
20–29	53 (21.0%)	13 (18.6%)	5 (12.5%)	7 (25.9%)	4 (8%)
30–39	94 (37.3%)	23 (32.9%)	16 (40.0%)	6 (22.2%)	15 (30%)
40–49	59 (23.4%)	11 (15.7%)	9 (22.5%)	7 (25.9%)	16 (32%)
50–59	40 (15.9%)	21 (30.0%)	10 (25.0%)	7 (25.9%)	14 (28%)
Over 60	6 (2.4%)	1(1.4%)	0	0	1 (2%)
**Tenure (Years):**	*n =* 253;	*n =* 70;	*n =* 41	*n*-28	*n =* 49;
0–9	59 (23.3%)	16 (22.9%)	5 (12.2%)	3 (10.7%)	5 (10.2%)
10–19	96 (37.9%)	33 (47.1%)	15 (36.6%)	14 (50.0%)	16 (32.6%)
20–29	59 (23.3%)	10 (14.3%)	12 (29.3%)	8 (28.6%)	17 (34.6%)
30–49	34 (13.4%)	8 (11.4%)	6 (14.6%)	2 (7.1%)	9 (18.3%)
Over 50	5 (2.0%)	1(1.4%)	3 (7.3%)	1 (3.6%)	2 (4.1%)
**Location of Work:**	*n =* 237;	*n =* 71	*n =* 42; 1 = 2 locations	*n*-28	*n =* 48;
Rural	19 (8.0%)	10 (14.1%)	13 (31.7%)	13 (46.4%)	4 (8%)
Urban	48 (20.3%)	14 (19.7%)	7 (17.1%)	3 (10.7%)	9 (18.7%)
Inner city	71 (30.0%)	14 (19.7%)	11 (26.8%)	4 (14.6%)	15 (31.2%)
Town	93 (39.2%)	31 (43.7%)	11 (26.8%)	12 (42.9%)	15 (31.2%)
Other location	6 (2.5%)	2 (2.8%)	0	2 (7.1%)	5 (10.4%)
**Hours Worked/Week:**	*n =* 240;	*n =* 67;	*n =* 41	*n =* 28	*n =* 50
0–20	6 (2.5%)	5 (7.5%)	1 (2.4%)	2 (7.1%)	1 (2%)
21–30	32 (13.3%)	12 (17.9%)	4 (9.8%)	2 (7.1%)	6 (12%)
31–40	205 (85.4%)	44 (65.7%)	36 (87.8%)	21 (75%)	38 (76%)
Over 40	5 (2.0%)	6 (8.9%)	0	3 (10.7%)	5 (10%)

**Table 2:** Summary of common tasks undertaken in three or more settings.

Task	Community	Hospital	Primary Care	GP Practice	Other
**Technical Tasks**					
Ordering/procurement inc. reconciliation and queries	Y	Y		Y	Y
Stock management	Y	Y		Y	Y
Order medicines for patients	Y	Y		Y	Y
Fridge management (e.g., temperature monitoring)	Y	Y		Y	
Dispensing	Y	Y		Y	Y
Accuracy Checking of Dispensed Items	Y	Y		Y	Y
Check endorsing on prescriptions	Y	Y	Y		Y
Maintain Accredited checking pharmacy technician (ACPT) competencies in dispensary	Y	Y		Y	
Dispensing compliance aids	Y	Y		Y	
Prescription admin (collection and filing, repeat supply)	Y	Y		Y	
Dispensing controlled drugs	Y	Y		Y	
Maintain legal registers	Y	Y	Y		
Assisting with audits	Y	Y	Y	Y	Y
**Clinical Tasks**					
Handing out medicines	Y	Y		Y	Y
Communication with multidisciplinary teams (MDT)	Y	Y	Y	Y	Y
Liaise with care/nursing homes	Y	Y	Y	Y	
General Communication (patients)	Y	Y	Y	Y	Y
Patient counselling	Y	Y	Y	Y	Y
Check allergies and interactions	Y	Y	Y	Y	
Medicines Optimisation (assisting with MURs, Drug history taking)	Y	Y	Y		
Healthy lifestyle advice (Essential Services, patient consultations)	Y	Y	Y		Y
**Management/Training Tasks**					
Preparing staff rotas and time sheets	Y	Y	Y	Y	Y
Organisational related activities e.g., figures to senior team	Y	Y	Y	Y	Y
Training and development (in-house training)	Y	Y	Y	Y	Y
Teaching/training pre-reg pharmacists. Pre and post reg pharmacy technicians, pharmacy assistants	Y	Y	Y		y
L2 assistant expert witnessing	Y	Y	Y		
Responding to queries via phone, email, face to face	Y	Y	Y		Y
Liaise with hospital, patients and community pharmacies	Y	Y	Y	Y	
Updating pharmacy IT systems	Y	Y	Y	Y	Y
Deal with complaints	Y	Y	Y	Y	Y

## Supplementary Materials

The following are available online at http://www.mdpi.com/2226-4787/5/3/40/s1, Table S1: Complete Task Table.
