# Supplementary material for: Defining the Role of the Pharmacy Technician and Identifying Their Future Role in Medicines Optimisation
Source: Pharmacy (Basel). 2017 Jul 15;5(3):40. doi: 10.3390/pharmacy5030040 (PMC5622352; doi:10.3390/pharmacy5030040)
Supplement: Supplementary file 1 [file pharmacy-05-00040-s001.pdf]

Supplementary Table S1. Complete task table

**Task**	**Community**	**Hospital**	**CCG**	**GP Practice**	**Education & Training**	**Other**
**Technical**						
Ordering/procurement (including invoice reconciliation & dealing with invoice queries)	y	y		y		y
Procurement contract monitoring		y				
Updating pharmacy IT systems	y	y	y	y	y	
Stock management	y	y		y		y
Ward stock top ups		y				
Fridge management (e.g. temperature monitoring)	y	y		y		y
Manage medicines waste		y	y			
Order medicines for patients	y	y		y		y
Dispensing	y	y		y		y
Accuracy Checking of Dispensed Items	y	y		y		y
Handing out medicines	y	y		y		y
Maintain ACPT competencies in dispensary	y	y		y		
Dispensing adherence aids	y	y		y		
Prescription administration (collection & filing, repeat supply)	y	y	y	y		
Communication (MDT)	y	y	y	y	y	y
General Communication (patients)	y	y	y	y		y
Dispensing controlled drugs		y		y		
Maintain legal registers	y	y	y			
Selling OTC	y	y				
Problem solving	y	y	y	y		y
Processing prescriptions for payment	y			y		
Eye clinic discharge prescriptions		y				
Clinical check of meds before faxing to satellite pharmacy (prison)						y
**Medicines Management**						
Medicines optimisation (assisting with MURs, drug history taking)	y	y	y			
Medicines Management (nursing homes)	y		y	y		

Patient home visits liaising with MDT teams including social workers and patient's families			y	y		
Check allergies and interactions		y	y	y		
Patient counselling (Handing out prescriptions)						
Follow up domiciliary visits with GPs			y			
Issue anticipatory Rxs for end of life care and arrange same day delivery				y		
MARS chart management			Y			
Compliance reviews in patients home			y			
Medicines optimisation (checking patients own drugs for use)		y	y			
Medicines optimisation (medicines reconciliation)		y	y	y		
Monitoring Clozapine		y				
Reconciling and ordering Clozapine for inpatients		y				
Complete medicines Reconciliation at off site rehab units for new admissions		y				
Visit community mental health team & provide support for safe and secure audits, FP10s		y				
Provide support to Clozapine & depot clinics: changes to doses and supplies		y				
Training on Antibiotics and infection control (MDT)		y			y	
Problem solving transfer of care of patients		y	y			
Warfarin counselling and training for staff		y	y			
Generate supply of meds for individual patients	y	y	y	y		y
Prescribing advice		y	y	y		y
Attend MMT and MDT meetings		y				y
Assist nurses with medicines administration (mental health)		y				
Discharge planning		y				y
Demonstrate use of appliance aids e.g. inhaler training		y	y			
Examples of clinical specialities (anticoagulant clinics, antimicrobial stewardship, renal/dialysis unit)		y	y	y		
Pastoral support with patients	y	y	y	y		y
Prepare discharge summary		y				
Undertake financial transactions	y	y		y		
Check MHRA Alerts	y					

Advising & liaising with Doctors on formulary medications and chart rewrites (prison)						y
Order pathology requests				y		
Managing warfarin administration by care workers including Point of Care testing for INR			y			
Return named CDs from ward		y				
Paediatric oncology trouble-shooter role		y				
Liaise between hospital, patients and community pharmacies regarding prescriptions	y	y	y	y		
Provide MI to a range of healthcare professionals including GPs		y				
Responding to queries via phone, email, face to face	y	y	y	y		
Provide pharmaceutical technical advice, support and information to care home staff		y				y
Liaising and communicating with MDT and patients, manufacturers	y	y	y		y	y
Updating supply issues for end of life medication	y					
Check endorsing on prescriptions	y	y	y			y
Co-ordinating across sectors for patient pathway (e.g. setting up patients with Hep b with community pharmacies,)	y	y	y	y	y	y
Providing healthy lifestyle advice (Essential services, patient consultations)	y	y	y	y	y	
Enhanced Services(e.g. palliative care service, minor ailments)					y	
Initiation of Audit, for example prescribing for asthma patients		y		y		
Data collection and management		y	y	y		
Assisting with audits	y	y	y	y	y	
Training on Antibiotics and infection control (MDT)		y				
Provide lunchtime learning sessions	y					
Frail and elderly patients referral		y	y			
Care home medication order reviews		y	y			y
Oncology clinic		y	y			
Travel advice		y	y			
**Organisational Management**						
Overseeing a service		y		y		y
Manage a regional Pharmacy Technician education Unit					y	

Staff management (appraisals, recruitment, return to work)		y	y			y
High level HR (e.g. disciplinary)		y	y	y		y
Budget control		y	y	y		y
Writing/review policies & procedures inc. SOPS		y	y		y	
Strategic planning (e.g. capacity issues, workload planning)		y				
Attending organisational meetings e.g. MDT		y	y			
Attending external meetings		y	y	y	y	
Chairing meetings		y	y			
Writing business cases		y				
Monitoring KPIs (HR)		y	y			
Preparing staff rotas & time sheets		y		y		
Accountable officer (CDs)			y			
Witness CD destruction by others e.g. nurses	y	y	y	y	y	
Controlled drugs (dispensing methadone and administration supervision))	y					
Report Trust drug spend			y			
Controlled drugs Destruction	y	y	y	y		y
Organisational related activities (supply figures to senior team)		y	y			y
**Training and Development**						
Training of care home staff to administer meds			y			
Training and development (in-house training)	y	y	y	y		y
Training other healthcare professions (ward based for nurses, OTs Physios)	y	y	y		y	
Lecturing for the Level 3 Diploma		y	y		y	
Supervise and train pre reg pharms and techs, marking work		y	y		y	
Undertake Assessment and verification for Level 4 Diploma		y	y		y	
Undertake NVQ Assessment	y	y	y		y	
Undertake IQA Verification		y	y		y	
Train prescription clerks			y			
Teaching/training pre-reg pharmacists/pharmacy technicians/pharmacy assistants in-house: MMS, Level 2 & 3		y	y	y	y	y
L2 assistant expert witnessing	y	y		y		
Facilitator for ACPT		y			y	
Training and assessing competency of support workers to administer medication						

Mentoring staff		y	y			y
Manage the quality assurance of education programmes		y			y	
Writing & updating training programmes		y	y		y	
**Clinical Governance**						
Quality assurance (error investigation and management)		y	y			
Compliance to medicines and CD policies		y	y			y
Datix/Incident reporting (investigating and reviewing)						
Participate in risk Management work relating to prescribing		y				
Lead on medicines management clinical governance issue relating to care homes, liaising with CCG Safeguarding Adults Lead and CQC			y			
Data analysis and writing reports (prescribing/incidents/usage and wastage and also for medicines management incentive schemes)			y			
**Manufacturing & Aseptics**		y				y
Checking of batches		y				y
Extemporaneous dispensing		y				
Manufacturing aseptic products		y				y
Check Customer service team orders (4 x a day)		y				
Calculate costs for chemotherapy prescriptions						
Health and safety risk assessments		y				y
Attend daily senior webex meetings						y
Building IT software						y
**Primary Care**						
Medicines Switches CCG incl letters to patients			y	y		y
Monitoring of Care Home with nursing prescribing incentive scheme			y			
Training carers on medication			y			
Admission avoidance team referrals			y			
Media campaigns			y			
Service Improvement advice			y			y
Regional drug contract amendments		y	y			
Review high cost drug data from acute trusts & identify anomalies			y			
Advise Care home staff on legal, safe and secure handling of medicines			y			
Admin/audit of Community Pharmacy Local enhanced services			y			

Coordinate patient and work load		y		y		
**Other**						
Academic research					y	
Research and clinical trials		y				
Project management/ project work		y	y		y	y
Visit Community mental health team	y					
Cleaning	y	y				
Monthly monitoring forms (company)	y					
Actioning tasks from lead pharmacist e.g. emergency drug boxes		y				
Adios reporting - providing evidence and justifying use of abusable drug usage on wards when usage increases						y
Weekly QT interaction lists for adaction teams (methadone)	y		y			
Perform user satisfaction survey		y				
Licensed and unlicensed quality review meetings		y				
Source new item requests		y				
Quality improvement work		y				
Error investigation		y				
Deal with complaints	y	y	y			
Spot checks on wards		y				
Mentoring staff		y				
Liaise with nursing staff re medication		y				
Process high cost medication requests		y				
Clear out medicines trolleys		y				
HIV clinic		y				
Represent department at external meetings		y	y			
Visit 4 main hospitals each week		y				
Haematology clinic		y				
Compile fridge data weekly		y				
Emergency cupboard supply		y				
Checking clozapine bloods		y				
Wastage reduction		y				
Environmental monitoring		y				

C Diff ward round		y				
Abx training for nurses		y				
Updating microguide app		y				
Formulary work		y				
Maintaining training databases		y				
Horizon scanning		y				
Vaccine storage and handling		y				
Read health protection bulletins			y			
Falls reviews		y	y			
Authorising staff payments on SSTS trials activities		y				
Home visits following discharge = adherence		y				
Answering Freedom of information requests			y			
Checking that medication is safe to administer		y	y			
Visits to patients at the request of GP practices and hospitals			y			
Train nurses, physios and OTs to check meds against Rx on transfer of care		y	y			
Collect prescribing data			y			
Respond to parliamentary questions						y
Respond to media questions						y
Represent HSCIC at industry exhibitions						y
